# Tympanoplasty—conchal cavum approach

**DOI:** 10.1186/s40463-015-0113-3

**Published:** 2016-01-06

**Authors:** S. Christopher Man, Desmond A. Nunez

**Affiliations:** Clinical Instructor, Division of Otolaryngology, Department of Surgery, University of British Columbia, 102-2620 Commercial Drive, Vancouver, BC Canada; Associate professor, Head / Division of Otolaryngology, Department of Surgery, University of British Columbia, Diamond Health Care Center, 2775 Laurel Street, Vancouver, BC Canada

## Abstract

**ᅟ:**

The three well recognized tympanoplasty approaches: permeatal, postaural, and endaural, each have advantages and disadvantages. The permeatal approach is suitable only for ears with adequate canal size. The postaural approach limits visualization of the posterior eardrum margin. The endaural approach limits the view of the eardrum's anterior margin.

This study describes a modified endaural approach, developed to overcome these limitations. A retrospective case series review and collection of a prospective cohort of patient reported outcome data were undertaken to assess the technique.

**Method:**

Standard incisions as used in an endaural approach are placed within the ear canal. The novel incision extends from the superior canal incision into the conchal cavum. This allows a flap of the thick, hairbearing skin from both the bony and cartilaginous portions of the canal to be raised, and everted, to provide an excellent view of the entire drum. Perichondrium can be harvested for grafting from the conchal cavum.

The clinical charts of all patients operated on by the first author using this technique from 2010–2012 were retrospectively reviewed. The size and position of the perforation, size of the canal, whether primary or revision surgery, graft take rate, hearing results and the occurrence of chondritis/perichondritis were recorded.

To investigate the morbidities and the acceptance by the patients of the incision/scar in the conchal cavum, all patients undergoing the procedure in the 8 months up to the end of August 2013 were prospectively recruited to complete a self-assessment Likert scale questionnaire recording postoperative pain, and satisfaction with the cosmesis of the operative site. The clinician recorded if there was any evidence of chondritis/perichondritis.

**Results:**

A 100 % graft take rate was achieved in the 75 adults treated by the first author from 2010 to 2012 regardless of the size and position of the perforation, configuration of the canal, primary or revision surgery.

Preoperative Pure Tone Audiometric (PTA) Air Bone Gap (ABG) averaged over 3 frequencies (0.5, 1 and 2 K Hz) was 19.4dB (standard deviation = 9.6, range 2 to 50). Postoperative PTA ABG average was 6.2 dB (standard deviation = 8.3, range -7 to 37), demonstrating a statistically significant post-surgery mean improvement of 13.2 dB (paired T-test, *p* < 0.001).

Twenty-one patients who underwent the procedure in 2013, reported minimal postoperative analgesic use, and scored the acceptability of the incision scar highly (4.8 out of a maximum of 5). There was no case of chondritis/perichondritis in the 96 cases.

**Conclusion:**

Whilst it is the surgeon’s decision to use a permeatal, postaural or endaural approach, the endaural approach with the conchal cavum modification is an excellent alternative to the traditionally described approaches.

**Trial Registration:**

Clinical trial number: NCT02000843 at ClinicalTrials.gov

## Introduction

There are three well recognised approaches utilized by surgeons when undertaking middle ear surgery namely the permeatal, postauricular, and endaural. These approaches are used in a range of operations where access to the ear drum and middle ear contents is necessary such as myringoplasty, tympanoplasty and stapedectomy. Each approach has advantages and disadvantages that combined with the individual surgeon’s preference for one technique over the others determines the approach used in any particular case. The permeatal approach restricts access to the tympanic membrane margins in patients with narrow ear canals. The post auricular approach requires a fair amount of soft tissue dissection with associated morbidity but provides favourable access to the eardrum’s anterior margin. The endaural approach limits access to the anterior eardrum margin.

## Objective

An improvement of the endaural approach is reported. An incision into the conchal cavum releases thick, hair bearing skin of posterior canal, allowing it to be moved out of the operative field, and brings the anterior eardrum margin into full view. Conchal perichondrium and/ or cartilage can be harvested by extending the conchal cavum incision. The details of the technique are described. The tympanoplasty graft take rate, hearing results, complications and patient satisfaction with the post-operative scar achieved in a series of patients using this modified approach are assessed and reported.

## Description of technique

### Skin incisions

#### Inside the canal

**Fig. 1:**
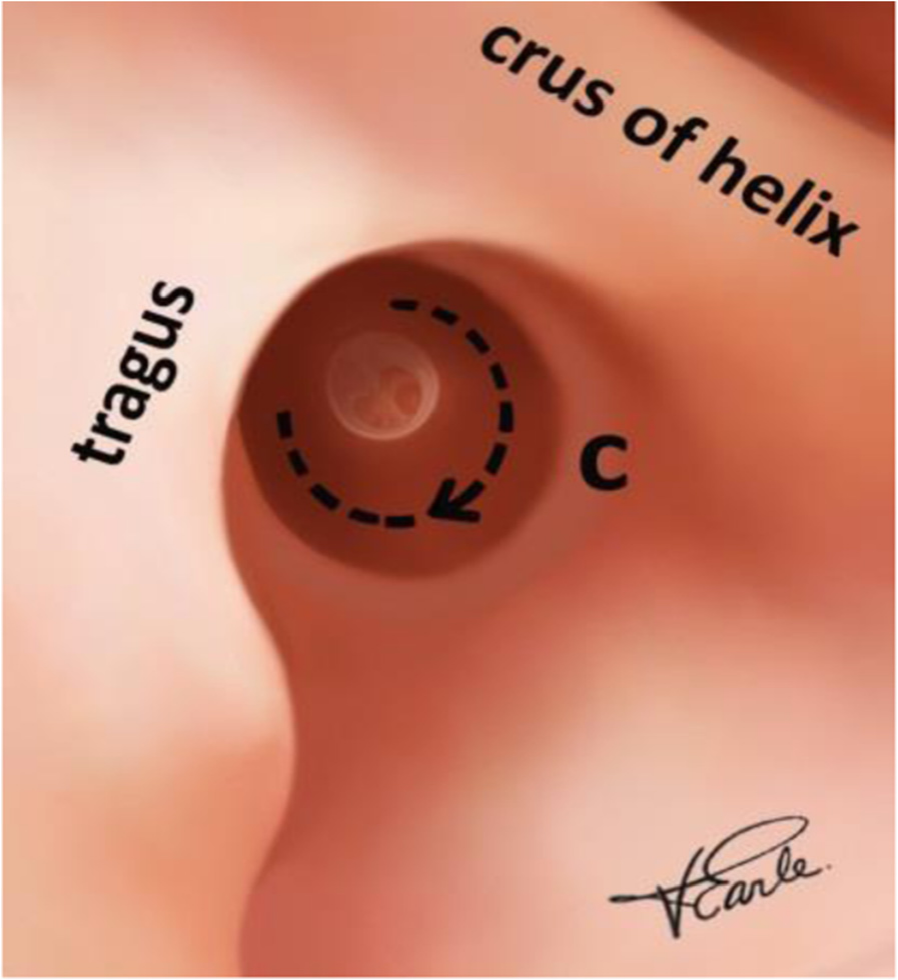
Circumferential in the ear canal incision. Legend- This illustrates the circumferential incision c. It is a half circle, 180°, on the posterior bony wall, which can be extended to 270° if indicated. It separates the thick hair bearing canal from the thin squamous epithelial lining

Standard incisions are used [1]. A 180 to 270° circumferential incision (incision c), starting just medial to the hairline of the external canal, extending from the 12 O’clock position posterior-inferiorly to the 6 O’clock position (180°) and when clinically indicated, continuing anteriorly to the 9 O’clock in a left ear (270°) divides the thick, hair-bearing skin from the thin, squamous epithelial lining of the canal (Fig. 1).

**Fig. 2:**
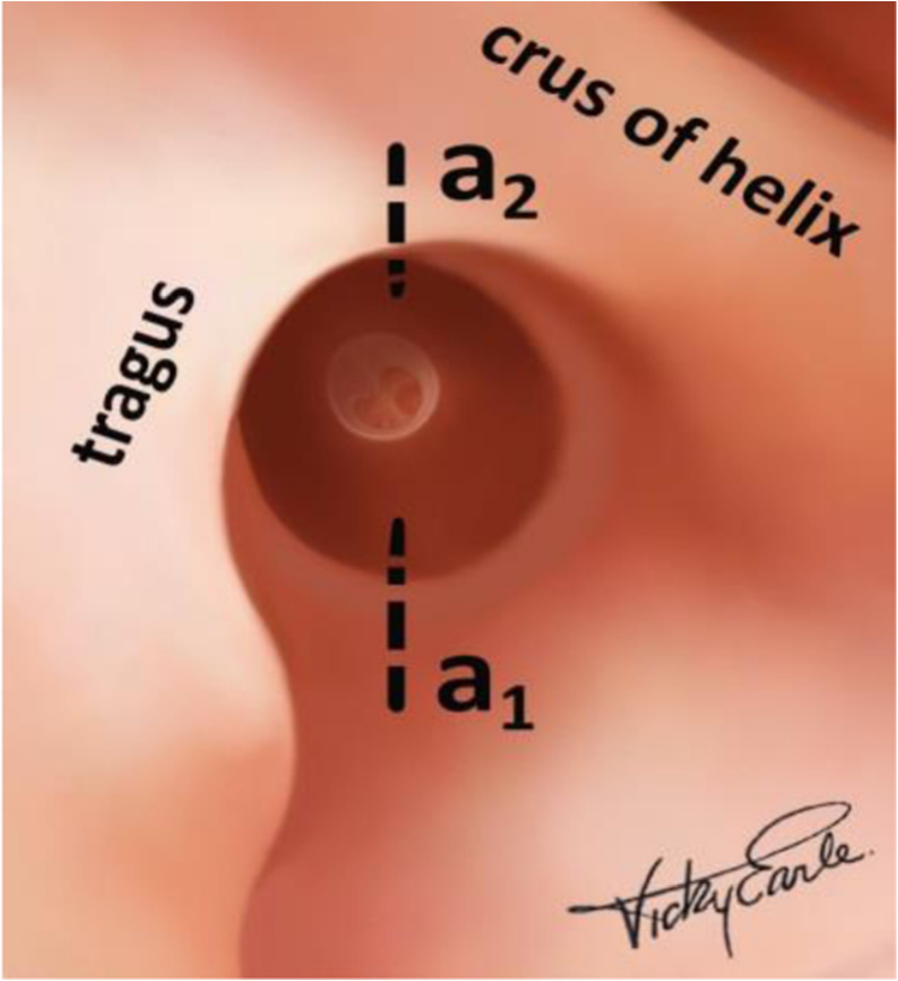
Radial ear canal incisions. Legend- The lower radial incision a1 and the upper radial incision a 2 are illustrated

At 6 and 12 O’clock, two lateral radial incisions, lower incision a1 and upper incision a2, are made (Fig. 2).

#### Outside the canal

**Fig. 3:**
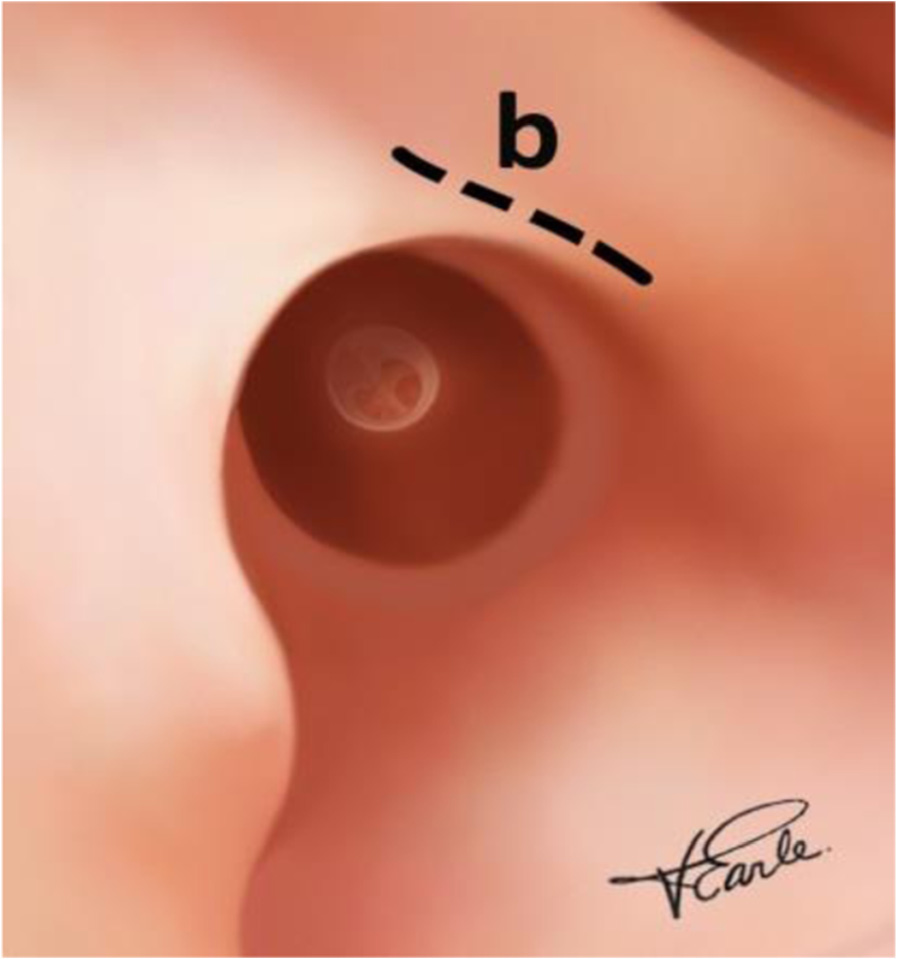
Conchal incision. Legend- The novel incision b, made at the superficial end of upper radial incision a2, extends into the conchal cavum, which allows eversion of the posterior canal skin

The novel incision, incision b, extends posteriorly from the superficial end of the upper radial incision (incision a2) into the conchal cavum inferior and parallel to the helix crus, for a total length of about 5 mm (Fig. 3).

The first author makes incisions a1, a2 first, followed by incision b and then incision c. However, the order is a matter of surgical preference.

### Dissection of the canal skin/squamous epithelium

The thick hairbearing skin from the both bony and cartilaginous portions of the canal posteriorly is dissected and elevated in a retrograde fashion.

**Fig. 4:**
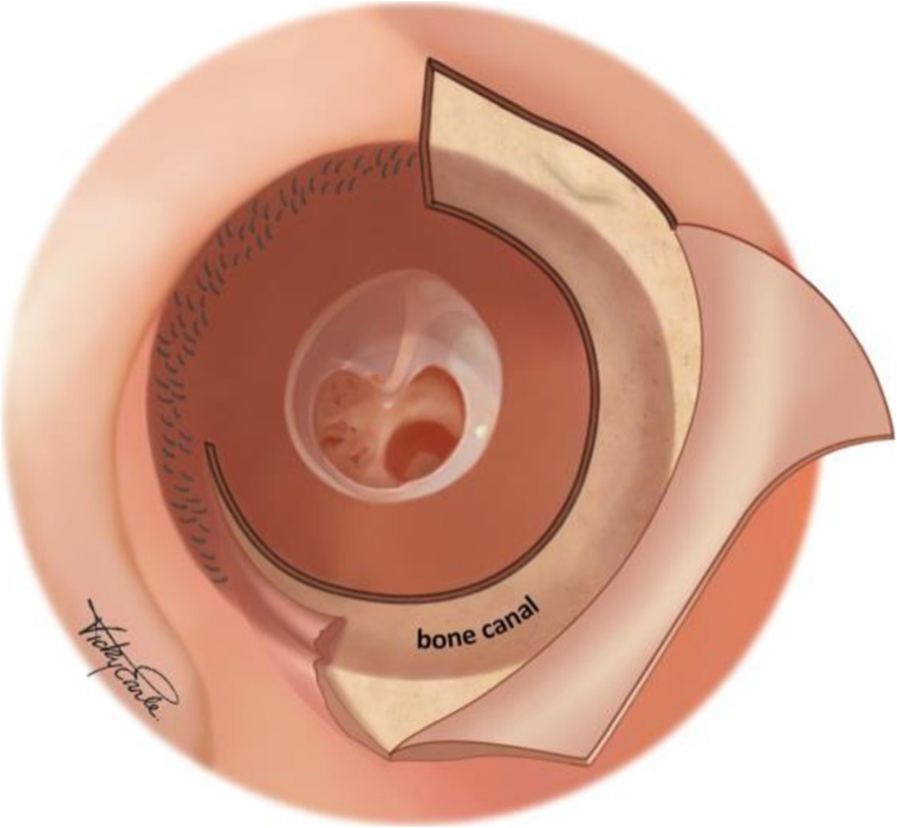
Bony ear canal exposure. Legend: The bony canal is exposed by everting the skin flap which consists of canal skin from both the bony and cartilaginous portions of the canal

The skin is turned inside out, exposing the posterior bony canal. With self-retaining retractor(s) in place, the canal skin is held away from the surgical field, fully exposing the bony ear canal (Fig. 4).

**Fig. 5:**
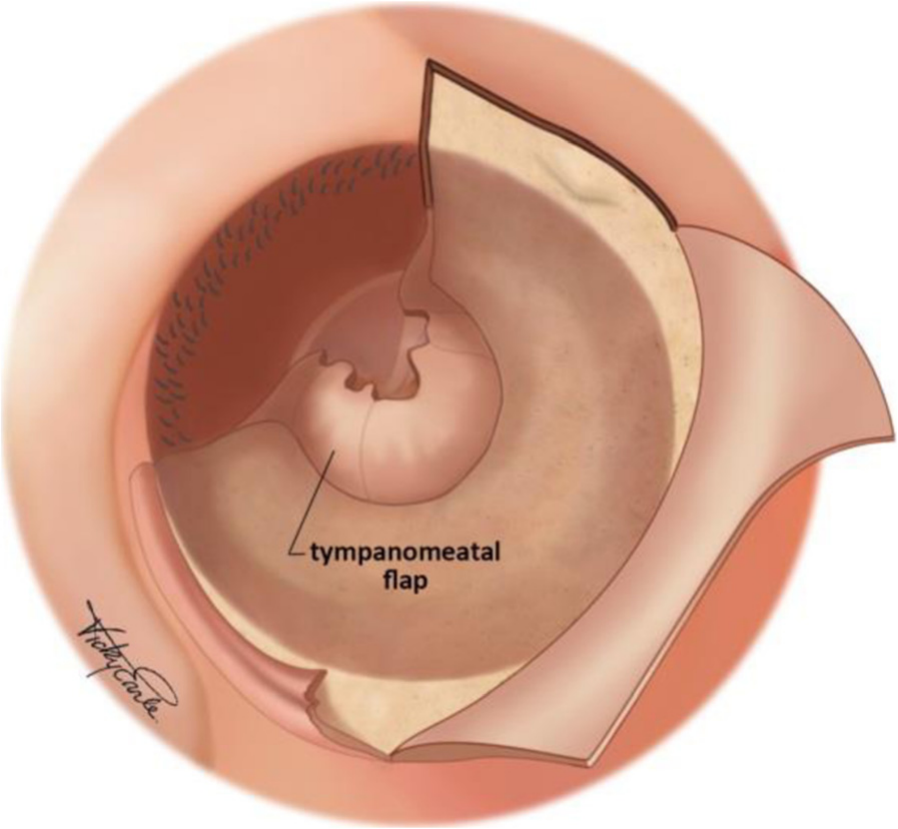
Cuff shape tympanomeatal flap. Legend: A cuff shape medial tympanomeatal flap is raised. The middle ear cavity can then be entered by lifting of the annulus from the tympanic ring

The cuff shape tympanomeatal flap of thin epithelial lining is elevated from the deep bony canal wall, and displaced medially (Fig. 5).

Any bony obstruction can be removed until the annulus is visualized, and elevated as required for underlay grafting.

### Harvesting of the graft

**Fig. 6:**
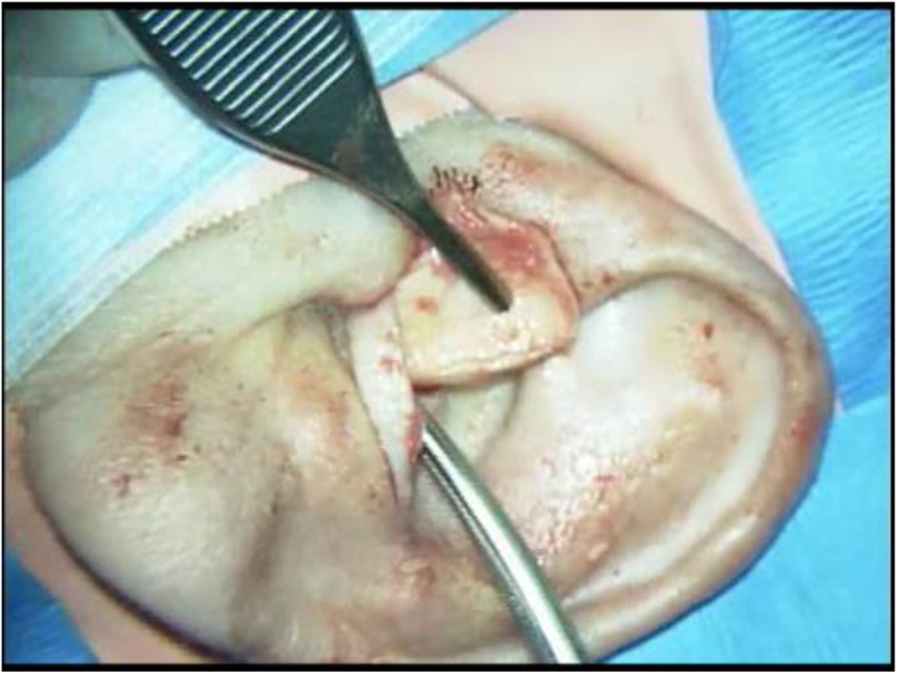
Conchal cartilage harvest. Legend: The harvesting of the conchal cartilage for grafting is shown

The conchal cavum incision is extended the full length of the upper border of the conchal cavum. The conchal cavum cartilage is dissected and harvested (Fig. 6). The perichondrium on the posterior surface is removed from the cartilage and used as grafting material. The cartilage, if not used, is returned to the conchal cavum.

### Patients and method

Approval for the research was granted by UBC-Providence Health Care Research ethics board. (H13-03261) Part A- The medical records of all patients who underwent conchal cavum approach tympanoplasty by the first author between 2010 and 2012 were reviewed to identify patients, who were followed up for a minimum of one year. No patient undergoing the procedure during the study period was excluded or lost to follow up. The review of records was undertaken in January 2014.

Data collected included the size and position of the perforations, size of the ear canal, if the procedure was primary or revision surgery, evidence of postoperative chondritis/perichondritis, pre- and post-operative Pure Tone Audiometric (PTA) Air and Bone Conduction hearing thresholds at 0.5, 1 and 2 KHz. The adequacy of the lumen of the external ear canal for surgical access was subjectively assessed at the time of surgery. A restricted ear canal was defined as one which precluded a full view of the ear drum margin using the operating microscope with the largest comfortable ear canal speculum in situ. The graft take rate at 12 months (complete closure of the perforation) was tabulated.

### Analysis

Averaged pre and post-operative Air Conduction (AC) and Bone Conduction (BC) were calculated. Individual patient's pre and post-operative Air Bone Gap (ABG) was calculated by substracting the averaged 3 frequency (BC from AC) thresholds recorded at the same sitting. Changes in conductive hearing loss were calculated according to the guidelines of the American Academy of Otolaryngology Head and Neck Surgery Committee on Hearing and Equilibrium (2), the only exception due to the retrospective nature of the data collection being the replacement of the 4 frequency with a 3 frequency average (0.5, 1, and 2K Hz).The Statistical Program for the Social Sciences (SPSS) version 23 was used to determine sample means, standard deviations and undertake a paried T test comparison of pre- and post-operative ABG for statistically significant differeence.

Part B - Patients undergoing the procedure between January and August 2013 were assessed at three weeks and three months postoperatively, recording postoperative analgesic use, conchal bowl appearance and numbness, occurrence of chondritis/perichondritis, and the patient’s satisfaction with the appearance of the conchal bowl and scar determined by a Likert scale questionnaire.

## Results

### Part A

Seventy-five patients (44 women and 31 men) treated from 2010 to 2012 meet the study inclusion criteria.

Their ages ranged from 23 to 75 years at the time of surgery. The age distribution by decade in this adult series was 3 aged 20–29, 10 aged 30–39, 19 aged 40–49, 28 aged 50–59, 13 aged 60–69, and 2 aged 70–79.

There were 29 subtotal or total perforations. 13 smaller perforations were anteriorly placed. The canal size was small or restrictive in 13 cases, 68 cases were primary, and 7 revision cases. All the perforations were completely healed on follow-up. There was no case of chondritis or perichondritis.

Preoperative PTA ABG averaged over 0.5, 1 and 2 K was 19.4dB (standard deviation = 9.6, range 2 to 50), and the Postoperative PTA ABG average was 6.2 dB (standard deviation = 8.3, range -7 to 37). There was a statistically significant mean 13.2 (standard deviation= 8.5, range -3 to 33) dB closure of the ABG with surgery (paired T-test, *p* < 0.001). 51, 16, 1, and 4 patients demonstrated a 0-10 dB, 11-20 dB, 21-30 dB, and >30 dB change in AB gap secondary to surgery respectively. In 4 patients the AB gap increased post surgery, in 2 by 2 dB and in 2 by 3 dB.

### Part B

Twenty-one patients who underwent a conchal approach tympanoplasty in 2013 were studied.

All patients were prescribed 1 to 2 tablets of Acetaminophen every 4 to 6 hourly as required for postoperative analgesia. 14 patients used Acetaminophen daily for one or two days. One patient used Acetaminophen for three days. One patient used 200 mg of Ibuprofen on the first post-operative day only. It is noteworthy that 5 patients did not use any postoperative analgesics.

No patients complained of numbness of the conchal bowl. The conchal bowl was well healed and retained its normal shape in all cases. There was no case of chondritis or perichondritis. On the Likert scale questionnaire, 5 patients agreed and 16 strongly agreed that the cosmesis of the conchal bowl and scar was excellent.

## Discussion

Good exposure of the annulus is important in underlay tympanoplasty. Obstruction of exposure by the cartilaginous and bony canal must be adequately addressed.

The skin of the cartilaginous canal and outer portion of bony canal is often thick and hair bearing. The bony canal is frequently curved, and narrow particularly at the isthmus.

With this conchal cavum approach, the thick hairbearing canal skin is mobilized and kept out of the way. The entire bony canal becomes easily accessible. Any bony obstruction interfering with the dissection of the annulus from the sulcus of the tympanic ring can be easily removed.

In the standard endaural approach, in order to gain more exposure, an incision is made between the tragus and helix, extending superiorly anterior to the crus of the helix [3]. A fair amount of dissection is needed. The exposure of the drum anteriorly is limited. Perichondritis from the pressure exerted by a retractor on the helical cartilage has been reported [4]. Also the scar in front of the helix is at times very visible.

In the conchal cavum approach, the incision in the conchal cavum releases soft tissues to be retracted without undue pressure and keeps it out of the way. It can be extended as far back as necessary to achieve a similar level of visualization of the drum and annulus anteriorly as achieved by the postaural approach. Usually, however, experience has shown only a short incision is necessary.

Even though the number of cases in this report is relatively small, it demonstrates that the approach can handle a variety of cases of differing canal size, perforation site and size. Revision cases were also successfully repaired with this technical modification.

The graft take rate compared well with reported graft take rates of 88 to 98.4 % [5, 6].

However, with more cases, it is anticipated that the graft take rate of 100 % will revert to the norm.

The hearing results also appeared to be in line with the literature of a range of 8 to 22 dB improvement [5, 6]. The use of this approach has not adversely affected the hearing results.

Postoperative morbidity was minimal. Cosmesis of the scar and conchal bowl was graded as excellent by the majority of the patients surveyed in this small sample. Many factors determine this judgment, and surgeons adopting this technique are advised to audit their own patients to ascertain their satisfaction with the cosmetic outcome. Postoperative pain was assessed indirectly through reported analgesic consumption, and whilst it is reasonable to conclude that the patients studied experienced little if any troublesome postoperative pain this was not a comparative trial. It is therefore not possible to determine if patients experienced less or more postoperative pain with this approach compared to other techniques. There were no cases of chondritis/ perichondritis, consistent with the literature reports of safe harvesting of cartilage and perichondrium from the concha [7-10].

The technique is not recommended for patients in whom keloid formation is a serious concern, though there were no such patients in this series.

## Conclusion

Whilst it is the surgeon’s decision to use a permeatal, postaural or endaural approach, the endaural approach with the conchal cavum modification is an excellent alternative to the traditionally described approaches.

## Abbreviations

PTA: pure tone audiometric
ABG: air bone gap
dB: decibel
AC: air conduction
BC: bone conduction
SPSS: statistical program for the social sciences
